# Proteome‐wide analysis of T‐cell response to BK polyomavirus in healthy virus carriers and kidney transplant recipients reveals a unique transcriptional and functional profile

**DOI:** 10.1002/cti2.1102

**Published:** 2020-01-14

**Authors:** George R Ambalathingal, Ross S Francis, Dillon Corvino, Sriganesh Srihari, Blake T Aftab, Corey Smith, Rajiv Khanna

**Affiliations:** ^1^ QIMR Berghofer Centre for Immunotherapy and Vaccine Development Tumour Immunology Laboratory QIMR Berghofer Medical Research Institute Herston QLD Australia; ^2^ Department of Nephrology Princess Alexandra Hospital Woolloongabba QLD Australia; ^3^ School of Medicine University of Queensland Brisbane QLD Australia; ^4^ Department of Preclinical and Translational Sciences Atara Biotherapeutics Los Angeles CA USA

**Keywords:** adaptive immunity, cellular immunity, immunology, immunotherapy, infectious diseases, innate immune cells, lymphocytes, T cells, viral infection

## Abstract

**Objectives:**

Cellular immunity against BK polyomavirus (BKV)‐encoded antigens plays a crucial role in long‐term protection against virus‐associated pathogenesis in transplant recipients. However, in‐depth understanding on dynamics of these cellular immune responses is required to develop better immune monitoring and immunotherapeutic strategies.

**Methods:**

Here, we have conducted a proteome‐wide analysis of BKV‐specific T‐cell responses in a cohort of 53 healthy individuals and 26 kidney transplant recipients to delineate the functional and transcriptional profile of these effector cells and compared these characteristics to T cells directed against cytomegalovirus, which is also known to cause significant morbidity in transplant recipients.

**Results:**

Profiling of BKV‐specific CD4^+^ and CD8^+^ T cells revealed that kidney transplant recipients with high levels of circulating viraemia showed significantly reduced T‐cell reactivity against large T and/or small T antigens when compared to healthy donors. Interestingly, T cells specific for these antigens showed strong cross‐recognition to orthologous JC virus (JCV) peptides, including those exhibiting varying degrees of sequence identity. *Ex vivo* functional and phenotypic characterisation revealed that the majority of BKV‐specific T cells from renal transplant recipients expressed low levels of the key transcriptional regulators T‐bet and eomesodermin, which was coincident with undetectable expression of granzyme B and perforin. However, *in vitro* stimulation of T cells with BKV epitopes selectively enhanced the expression of T‐bet, granzyme B and cellular trafficking molecules (CCR4, CD49d and CD103) with minimal change in eomesodermin and perforin.

**Conclusions:**

These observations provide an important platform for the future development of immune monitoring and adoptive T‐cell therapy strategies for BKV‐associated diseases in transplant recipients, which may also be exploited for similar therapeutic value in JCV‐associated clinical complications.

## Graphical abstract

This manuscript provides comprehensive profiling of BK polyomavirus T‐cell responses in healthy virus carriers and kidney transplant recipients.

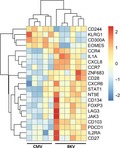

## Introduction

BK polyomavirus causes clinical complications such as BK virus‐associated nephropathy (BKVAN) and ureteral stenosis in kidney transplant recipients, and haemorrhagic cystitis in human stem cell transplant and bone marrow transplant patients.^1^, ^2^, ^3^ Reactivation of BKV leading to BKV viraemia is identified in about 10% of renal transplant recipients. A proportion of these patients will develop BKVAN, of which 90% lose their graft within a year.^1^, ^2^, ^3^ While in immunocompetent individuals BKV has not been associated with any clinical illness, impairment of immune responses in transplant recipients due to underlying immunosuppression contributes to the reactivation of latent BKV infection.^4^, ^5^ Reduction in the levels of immunosuppressive therapy upon the detection of viral load above 1 × 10^4^ copies per mL in plasma or urine is the preferred therapeutic approach for clinical management of BKV reactivation.^6^ As with many other latent viral infections, reduction in immunosuppression can facilitate the reconstitution of T‐cell immunity which can reduce the risk of developing BKVAN.^6^ However, broader knowledge of T cell‐mediated immune regulation of BKV infection remains rather limited.

BK polyomavirus infection is acquired early in childhood and can persist lifelong. T‐cell immune responses against BKV play a crucial role in controlling persistent infection. It is known that more than 90% of the population are seropositive for BKV.^7^ Despite the likely importance of T cells in controlling persistent infection in immunocompetent individuals, comprehensive analysis of T‐cell immunity to BKV in a large cohort of volunteers has not been performed. Hence, a study on the T‐cell response to BKV antigens in healthy individuals and transplant recipients could help in profiling the function of BKV‐specific T cells and promote the development of novel strategies to treat BKV‐associated diseases.

Previous studies have primarily focused on T‐cell responses restricted through the HLA‐A*02 allele in kidney transplant patients and healthy individuals.^8^, ^9^ A very limited number of studies have been conducted to define T‐cell responses restricted through a more broadly applicable set of HLA alleles, and the epitopes recognised by CD4^+^ T cells have similarly been rarely studied. The current study focuses on comprehensive profiling of BKV‐specific T‐cell responses to the three major capsid proteins (VP1, VP2 and VP3) and two viral replication proteins (LTA and STA) in healthy immunocompetent individuals and kidney transplant recipients. Subsequent analysis in this study focuses on comprehensive mapping of BKV‐specific T‐cell epitopes and determination of cognate HLA restrictions. To determine the relative prevalence of peptide epitopes of varying amino acid lengths in CD8^+^ and CD4^+^ T‐cell recognition, this study utilised overlapping peptides in combination with functional assays rather than Web‐based algorithms to predict epitopes. Twenty CD8^+^ T‐cell epitopes (restricted through HLA class I alleles) and 10 CD4^+^ T‐cell epitopes (restricted through HLA class II alleles) were identified during this study. With this knowledge of T‐cell epitopes, we further characterised the immunological features of BKV‐specific T cells, both in healthy individuals and in kidney transplant recipients.

## Results

### Kidney transplant patients with high viraemia show altered T‐cell reactivity against BKV‐encoded antigens

**Figure 1:**
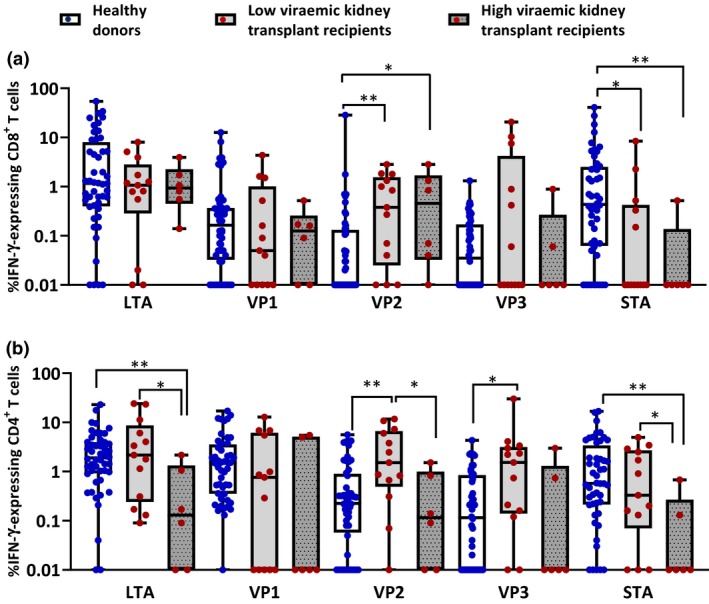
Profiling of BKV‐specific T‐cell responses in healthy individuals and kidney transplant recipients. PBMC from 53 healthy donors and 26 kidney transplant recipients (17 low viraemic and 9 high viraemic) were assessed for BKV‐specific T‐cell immunity against LTA, VP1, VP2, VP3 and STA antigens. PBMC were stimulated with overlapping peptide pools (OPPs) from each BKV‐encoded antigen, and antigen‐specific T cells were expanded for 14 days in the presence of IL‐2. Following *in vitro* expansion, these T cells were assessed for IFN‐γ expression using ICS assay on day 14 following stimulation with respective peptide pools. Panels **a** and **b** show comprehensive analysis of BKV‐specific CD8^+^ and CD4^+^ T cells, respectively. Statistical significance across multiple comparisons was determined using nonparametric Wilcoxon *t*‐test (*P‐value < 0.0313; **P‐value < 0.0021).

To comprehensively profile BKV‐specific T‐cell responses, we recruited 53 healthy individuals and 26 kidney transplant recipients with a history of BKV reactivation. Previous observations have indicated that BKV‐specific T‐cell responses are difficult to detect directly *ex vivo* in peripheral blood mononuclear cells (PBMC).^10^, ^11^ In concordance with these previous reports, the frequency of BKV‐specific T cells in PBMC was below detectable limits when intracellular cytokine staining (ICS) analysis was used for immune profiling (data not shown). To enhance the sensitivity of detection of BKV‐specific T cells, PBMC from healthy individuals and kidney transplant recipients were stimulated with proteome‐wide BKV overlapping peptide pools (OPPs) and cultured for 14 days in the presence of IL‐2 and T‐cell growth factor (TCGF). BKV specificity of these cultured T cells was then assessed using an ICS assay. This analysis clearly showed that CD8^+^ T‐cell responses in healthy individuals were predominantly directed towards LTA and STA, while VP1, VP2 and VP3 antigens were comparably less frequently recognised (Figure 1a). CD4^+^ T‐cell responses in healthy individuals were predominantly directed towards LTA, VP1 and STA (Figure 1b). Extension of BKV‐specific T‐cell profiling to kidney transplant recipients revealed that patients with viral load of >1 × 10^3^ copies per mL in plasma (referred to as high viraemic recipients) had significantly reduced CD8^+^ and CD4^+^ T‐cell reactivity against STA and/or LTA antigens when compared to healthy individuals (Figure 1a and b). Interestingly, kidney transplant recipients with viral load <1 × 10^3^ copies per mL of plasma (referred to as low viraemic recipients) showed significantly increased CD4^+^ T‐cell reactivity against VP2 and VP3 antigens when compared to healthy donors (Figure 1b). Furthermore, kidney transplant recipients with high and low viral load showed significantly increased CD8^+^ T‐cell reactivity against VP2 antigen (Figure 1a). Taken together, these analyses clearly showed that active BKV reactivation in kidney transplant patients alters the T‐cell reactivity against virally encoded antigens.

**Figure 2:**
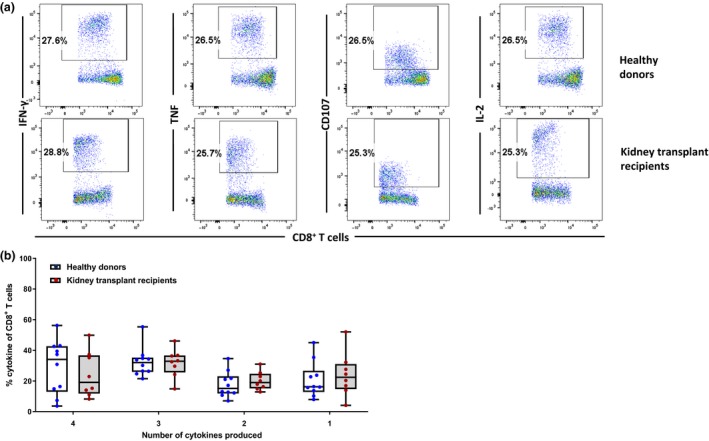
Polyfunctional profile of BKV‐specific T cells in healthy individuals and kidney transplant recipients. **(a)** Representative flow cytometry plots showing expression of IFN‐γ, TNF, IL‐2 or CD107a in BKV‐specific T cells from healthy virus carriers and kidney transplant recipients. **(b)** Boolean analysis to determine the polyfunctionality (CD107a, IFN‐γ, TNF and/or IL‐2) of the BKV‐specific T cells from healthy individuals and kidney transplant recipients (*n* = 10 for healthy individuals and *n* = 8 for kidney transplant recipients).

Previous studies have highlighted the importance of the polyfunctional profile of antigen‐specific T cells and its impact on disease outcomes.^12^, ^13^, ^14^ To further expand the functional profiling of BKV T cells, we assessed polyfunctional capability of antigen‐specific T cells from both healthy individuals and kidney transplant recipients. *In vitro* expanded BKV‐specific T cells were assessed for the production of IFN‐γ, TNF, CD107a and IL‐2 by intracellular cytokine staining following stimulation with HLA class I‐restricted BKV‐specific T‐cell epitopes (Figure 2a). Analysis of the polyfunctional profile comparing the number of cytokines produced by responding T cells displayed no significant differences (Figure 2b).

## Fine mapping of CD8^+^ and CD4^+^ T‐cell epitopes within BKV‐encoded proteins

**Figure 3:**
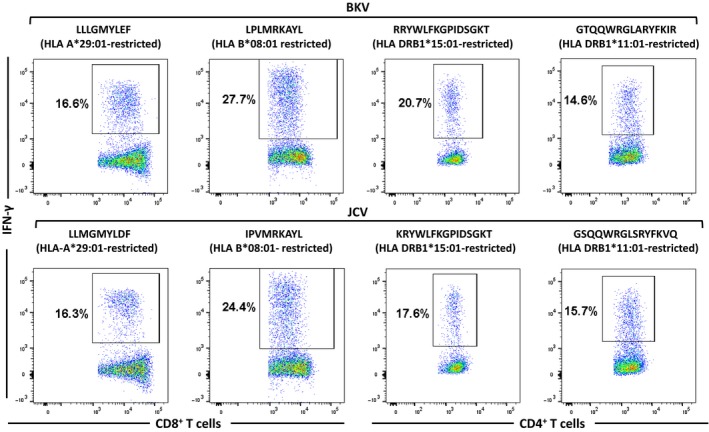
Cross‐recognition of BKV and JCV peptides by BKV‐specific T cells. Representative flow cytometry plots showing IFN‐γ expression by BKV‐specific CD8^+^ and CD4^+^ T cells following stimulation with HLA class I‐ and class II‐restricted BKV epitopes and homologous JCV peptides.

Based on the profiling of *in vitro* expanded BKV‐specific T‐cell responses, we selected LTA, STA and VP1 antigens for precise mapping of HLA class I‐ and class II‐restricted T‐cell epitopes within BKV‐encoded proteins. Epitope mapping was conducted using overlapping peptides (15‐aa‐long overlapping by 10 aa) distributed within a two‐dimensional peptide matrix, creating a concerted series of small overlapping peptide pools.^15^ These matrices are designed so that each peptide occurs once on the ordinate.^16^ These peptide pools were used in a standard ICS assay for identifying the individual reactive 15‐mer peptides. Further minimisation of the epitope sequence was then carried out to identify the optimal T‐cell epitope sequence for each identified 15‐mer. The 15‐mer peptide sequences were trimmed iteratively from both n‐ and c‐terminus to final minimal 9‐aa‐long peptides. Representative dot plots of T‐cell responses for three novel CD8 and CD4 T‐cell epitopes in both healthy individuals and transplant recipients are shown in Supplemental figure 1. Following minimisation, resulting 9‐mer peptides were tested against T cells in a standard ICS. HLA restriction of the epitope was then identified by stimulating T cells using peptide‐loaded HLA‐matched and HLA‐mismatched lymphoblastoid cell lines. A comprehensive list of CD8^+^ and CD4^+^ BKV T‐cell epitopes and their HLA restriction are listed in Tables 1 and 2. This analysis showed that the list of epitopes mapped during this study could cover 96.8% of the world population and 98.15% and 92.4% of the population in the United States and Australia, respectively. We further extended this analysis to explore the potential cross‐reactivity of BKV‐specific T cells to orthologous JC virus (JCV)‐encoded antigens. This analysis showed that 14/20 (70%) and 8/10 (80%) BKV CD8^+^ and CD4^+^ T‐cell epitopes showed cross‐reactivity with JCV peptide sequences, respectively (Tables 1 and 2). Representative data presented in Figure 3 show multiple examples of BKV‐specific T cells cross‐recognising JCV peptide sequences.

**Table 1:** List of CD8^+^ T‐cell epitopes, HLA restriction, frequency and cross‐reactivity

Peptide sequence	HLA	No. of donors tested	No. of donors who responded	JCV variant sequence	JCV cross‐reactivity
NREESMELMDL	B*40:01	6	1	NREESMELMDL	Yes
MELMDLLGL	B*40:01	6	2	MELMDLLGL	Yes
LPLMRKAYL	B*07:01	9	7	IPVMRKAYL	Yes
LPLMRKAYL	B*08:01	7	4	IPVMRKAYL	Yes
SQHSTPPKK	A*11:01	7	1	SQHSTPPKK	Yes
TPHRHRVSA	B*56	3	2	TPHRHRVSA	Yes
VFLLLGMYLEF	A*23:01	3	1	VFLLMGMYLDF	Yes
AVDTVLAKK	A*11:01	7	4	AVDTVAAKQ	No
FPLCPDTLY	B*35:01	7	2	FPPNSDTLY	No
FPLCPDTLYC	B*57:01	4	1	FPPNSDTLYC	No
VHCPCMLCQL	B*39	1	1	VHCPCLMCML	Yes
EPLVWIDCY	B*35:01	7	3	SPLVWIDCY	Yes
CYCIDCFTQW	A*24:01	4	1	CYCFDCFRQW	No
LLIKGGVEV	A*02:01	7	1	LLIRGGVEV	No
AITEVECFL	A*02:01	7	3	SITEVECFL	Yes
NLLMWEAVTV	A*02:01	7	4	NILMWEAVTL	Yes
FFAVGGDPLEM	B*40:01/06	6	2	FFSVGGEALEL	No
LLLGMYLEF	A*29:01	3	1	LLMGMYLDF	Yes
ARIPLPNL	B*27:07	2	1	ARIPLPNL	Yes
YCIDCFTQW	B*57:01	4	1	YCFDCFRQW	No
IEESIQGGL	B*40:01	6	2	VEESIQGGL	Yes

**Table 2:** List of CD4^+^ T‐cell epitopes, HLA restriction, frequency and cross‐reactivity

Peptide sequence	HLA	No. of donors tested	No. of donors who responded	JCV variant sequence	JCV cross‐reactivity
GTQQWRGLARYFKIR	DRB1*11	6	4	GSQQWRGLSRYFKVQ	Yes
RGLARYFKIRLRKRS	DRB1*11	6	1	RGLSRYFKVQLRKRR	Yes
RKAYLRKCKEFHPDK	DRB1*13	6	2	RKAYLKKCKELHPDK	Yes
WDEDLFCHEDMFASD	DQB5*01	3	1	WDEDLFCHEEMFASD	No
CFTQWFGLDLTEETL	DRB1*03/04	5	2	CFRQWFGCDLTQEAL	Yes
GGDEDKMKRMNTLYK	DRB1*13	8	4	GGDEDKMKRMNFLYK	Yes
KMKRMNTLYKKMEQD	DRB1*13	8	2	KMKRMNFLYKKMEQG	Yes
FNVPKRRYWLFKGPI	DRB1*15	5	2	LNIPKKRYWLFKGPI	Yes
RRYWLFKGPIDSGKT	DRB1*15	5	2	KRYWLFKGPIDSGKT	Yes
VGPLCKADSLYVSAA	DRB1*07	5	2	VGPLCKGDNLYLSAV	No

### BKV‐specific T cells display a less differentiated early‐effector T‐cell profile

**Figure 4:**
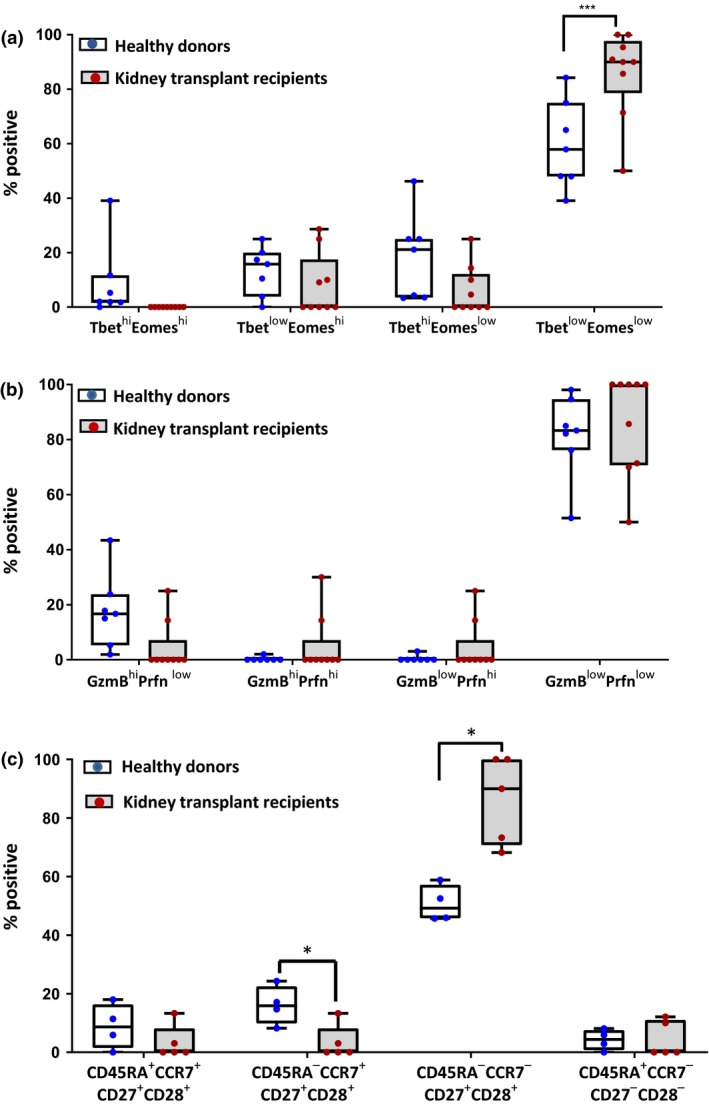
*Ex vivo* transcriptional, effector molecule and phenotypic profiling of BKV‐specific T cells. BKV‐specific T cells were enriched from the PBMC of healthy individuals and kidney transplant recipients using MHC–peptide dextramers and magnetic beads. The enriched cells were labelled with antibodies specific for individual markers and then analysed by flow cytometry. **(a)** Expression of transcription factors T‐bet and Eomes in BKV‐specific T cells from healthy individuals (*n* = 7) and kidney transplant recipients (*n* = 8). **(b)** Expression of cytolytic effector molecules including granzyme B and/or perforin in healthy individuals and kidney transplant recipients. **(c)** The immunophenotyping of BKV‐specific T cells using antibodies specific for CD27, CD28, CCR7 and CD45RA. The combinatorial analysis of the expression of these markers in BKV‐specific T cells was categorised as CD45RA^+^CCR7^+^CD27^+^CD28^+^, CD45RA^−^CCR7^+^CD27^+^CD28^+^, CD45RA^−^CCR7^+/−^CD27^+^CD28^+^ or CD45RA^+^CCR7^−^CD27^−^CD28^−^. Statistical significance across multiple comparisons was determined using nonparametric Wilcoxon *t*‐test (*P‐value < 0.0313; ***P‐value < 0.002).

Transcription factors T‐bet and Eomes play a key role in the differentiation of T cells,^17^, ^18^ and their differential expression influences the effector profile of T cells.^17^, ^18^, ^19^ To characterise the expression of T‐bet and Eomes in BKV‐specific T cells, we developed an enrichment protocol to isolate BKV‐specific T cells from the PBMC of healthy individuals and renal transplant recipients. PBMC were labelled with MHC–peptide dextramers conjugated to a fluorochrome, and then, magnetic beads coated with anti‐fluorochrome antibody were used to positively select the antigen‐specific cells using magnetic enrichment (Supplemental figure 2). These enriched cells were then assessed for the expression of transcription factors and effector molecules. BKV‐specific T cells from healthy individuals displayed a predominant T‐bet^low^Eomes^low^ and granzymeB^low^perforin^low^ phenotype (Figure 4a and b). Kidney transplant patients displayed a similar profile; however, there was a significant increase in the proportion of T‐bet^low^Eomes^low^ T cells (Figure 4a). To further delineate the characteristics of BKV‐specific T cells, MHC–peptide dextramer‐enriched cells were co‐stained for T‐cell surface markers that can be used to classify T cells into effector and memory subsets. BKV‐specific cells were labelled with antibodies specific for CD27, CD28, CD45RA and CCR7. It was noted that BKV‐specific T cells from both healthy individuals and kidney transplant recipients had high expression of early‐effector or central memory markers including CD27 and CD28, and lower expression of the late‐effector marker, CD45RA. Combinatorial analysis of phenotypic marker expression revealed that BKV‐specific T cells from kidney transplant recipients were predominantly CD45RA^−^CCR7^−^CD27^+^CD28^+^, while these antigen‐specific T cells from healthy individuals included both CD45RA^−^CCR7^−^CD27^+^CD28^+^ and CD45RA^−^CCR7^+^CD27^+^CD28^+^ populations (Figure 4c).

## BKV‐specific T cells show differential expression of key immune modulatory genes compared to CMV‐specific T cells

**Figure 5:**
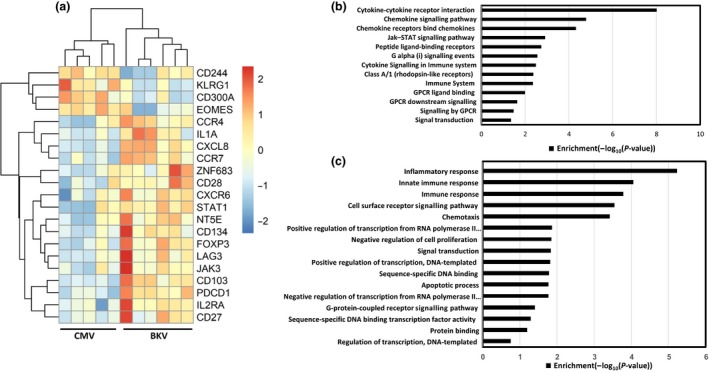
Gene expression analysis of BKV‐ and CMV‐specific T cells. *In vitro* expanded BKV‐ and CMV‐specific T cells were enriched using antigen‐specific dextramers (*n* = 5 for CMV‐specific T cells and *n* = 6 for BKV‐specific T cells). These sorted cells were lysed and used for gene expression analysis using NanoString nCounter gene expression platform. **(a)** Expression heatmap of significantly differentially expressed genes (*t*‐test corrected *P* < 0.05) between CMV‐ and BKV‐specific T cells. **(b)** Significantly enriched KEGG and Reactome pathways among the differentially expressed genes (*P* < 0.01). **(c)** Significantly enriched Gene Ontology terms – Molecular Function and Biological Process – among the differentially expressed genes (*P*‐value < 0.01).

To further characterise BKV‐specific T cells, we assessed the molecular expression of various immune regulating molecules in BKV‐specific T cells using the NanoString nCounter gene expression platform.^20^ A total of 136 genes involved in various immune mechanisms were included in the probe set, allowing the investigation of the immune characteristics of BKV‐specific T cells.^21^ Gene expression profile of BKV‐specific T cells from healthy individuals was contrasted with T cells directed against another persistent viral infection, cytomegalovirus (CMV), which is also associated with significant morbidity in transplant recipients. BKV‐ and CMV‐specific T cells were expanded in the presence of IL‐2 for 14 days, labelled with MHC–peptide dextramers and sorted using fluorescence‐activated cell sorting. The lysate of these cell populations was then used for gene expression analysis. Twenty‐one significantly differentially expressed (DE) genes (corrected *P*‐value < 0.05) were identified between BKV‐ and CMV‐specific T cells (Figure 5a). These differentially expressed genes included key transcription factors (T‐bet, Eomes, FoxP3, GATA), effector molecules (IL‐8, granzyme B, perforin), homing markers (CCR4, CD103, CD18, CD11a) and cell surface receptors (CD28, CD27, CD45RA, CCR7). Pathway analysis of these DE genes identified significant enrichment for cytokine–cytokine receptor interaction, chemokine signalling, JAK–STAT signalling and immune‐system‐related pathways (Figure 5b). The participation of the genes in these pathways was further confirmed by their significant enrichment for functions/processes including inflammatory response, innate immune response and cell surface receptor signalling (Figure 5c).

**Figure 6:**
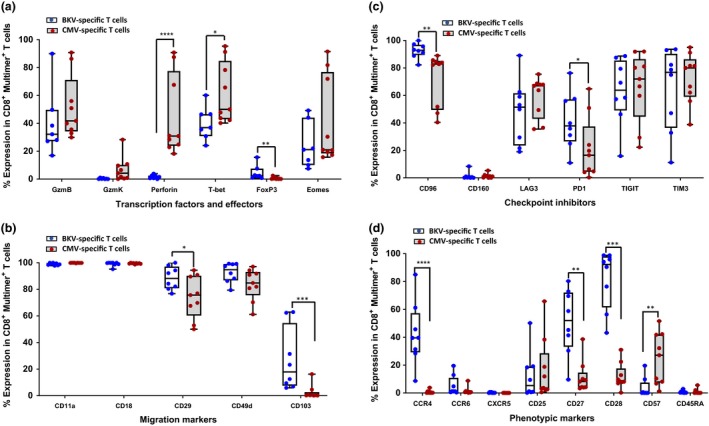
Immune profiling of BKV‐ and CMV‐specific T cells. **(a)** Comparative analysis of expression of cytolytic molecules (GzmB, GzmK and perforin) and transcription factors (T‐bet, Eomes and FoxP3) in BKV‐ and CMV‐specific T cells. **(b)** Expression of cell migration and homing markers (CD11a, CD18, CD29, CD49d and CD103) in BKV‐ and CMV‐specific T cells. **(c)** Expression of checkpoint inhibitor molecules (CD96, CD160, LAG3, PD1, TIGIT and TIM3) in BKV‐ and CMV‐specific T cells. **(d)** Expression of CCR4, CCR6, CXCR5, CD25, CD27, CD28, CD57 and CD45RA in BKV‐ and CMV‐specific T cells. The immune profiling analysis was done using BKV‐specific T cells from eight healthy donors and CMV‐specific T cells from nine healthy donors. Statistical significance across multiple comparisons was determined using nonparametric Wilcoxon *t*‐test, where *P‐value < 0.033, **P‐value < 0.0021, ***P‐value < 0.0002 and ****P‐value < 0.0001.

Further validation of these differentially expressed individual genes was confirmed at protein level using marker‐specific antibodies (Figure 6a–d; Supplementary figure 3). Consistent with the observations in our gene expression analysis, we observed significant differences in the expression of CD103 and CCR4 (higher in BKV‐specific T cells) and perforin (lower in BKV‐specific T cells) (Figure 6a and b). We also observed trend towards differential expression in PD1 (higher in BKV‐specific T cells), and granzyme K and B, Eomes and T‐bet (lower in BKV‐specific T cells) (Figure 6c). We also noted significant differences in the expression of other phenotypic markers (CD28, CD27 and CD57) that were not evident in gene expression analysis, but are consistent with other differences in the phenotypic and gene expression profile of BKV‐ and CMV‐specific T cells (Figure 6d).

**Figure 7:**
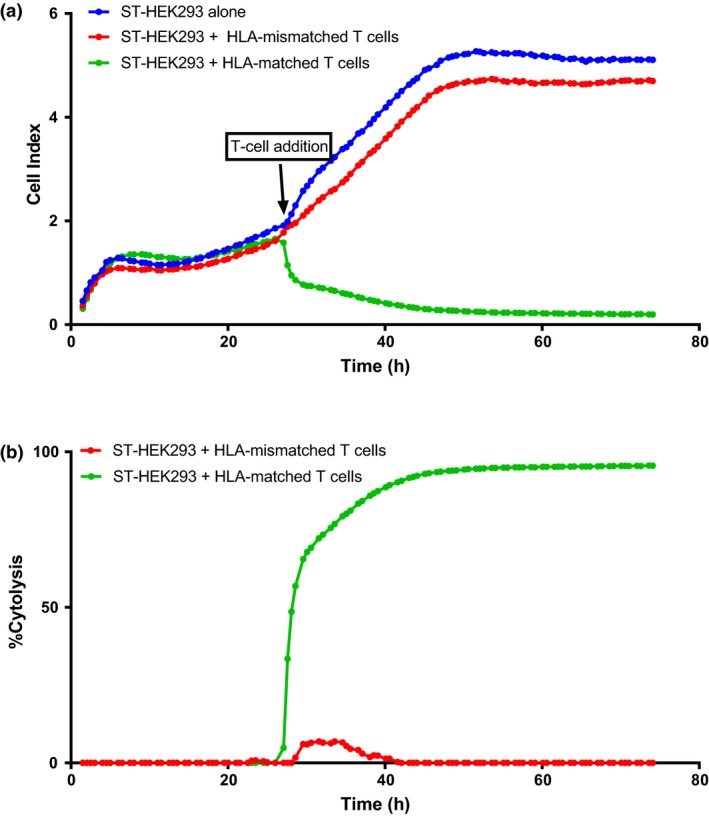
Cytolysis of target cells expressing BKV‐encoded STA protein by HLA‐matched BKV‐specific T cells. HEK293 cells (HLA B7^+^) expressing BKV‐encoded STA protein (ST‐HEK293) were exposed to HLA B7‐restricted BKV‐specific CD8^+^ T cells at an effector‐to‐target ratio of 10:1. HLA‐mismatched T cells were used as control in these assays. ST‐HEK293 cells (6 × 10^4^) were seeded in E‐plate, and the proliferative index of the cells was acquired in the RTCA system for 24 h. The T cells were added to the appropriate cells, and the data were acquired for 72 h. **(a)** The proliferative cell index for the target cells alone (ST‐HEK293) is shown by the blue line, that of the target cells added with HLA‐mismatched T cells is represented by the red line, and that of the target cells added with HLA‐B7‐restricted BKV‐specific T cells is represented by the green line. **(b)** Cytolysis of ST‐HEK293 was calculated based on the RTCA system specification and is represented as a line graph where the red line is the percentage lysis of target cells with HLA‐mismatched T cells while the green line is the percentage lysis of target cells with specific T cells.

These observations suggested that BKV‐specific T cells might have reduced cytolytic capacity due to relatively lower expression of effector molecules (e.g. perforin). To assess the cytolytic capacity of BKV‐specific T cells, we established a real‐time *in vitro* T‐cell killing assay using the xCELLigence RTCA platform. This assay allows assessment of target cell killing over a period of 72 h. HLA B7^+^ HEK293 cell line stably expressing BKV STA protein was used as target, and HLA B7‐restricted CD8^+^ T cells directed against STA were used as effectors. HLA‐A*11:01‐restricted BKV‐specific T cells were used as HLA‐mismatched control. The addition of the HLA B7‐restricted T cells induced a dramatic drop in the cell proliferation index of the ST‐HEK293 cells (Figure 7a), which translated to near 100% cytolysis of the ST‐HEK293 cells by 40 h (Figure 7b). In contrast, little evidence of growth inhibition or cytolysis was evident following the addition of the HLA‐mismatched T cells (Figure 7a and b).

## Discussion

The incidence of BKV‐associated diseases has been increasing over the last decade following the introduction of increasingly potent immunosuppression regimens in solid organ and stem cell transplant patients.^22^ It has become apparent that the increased risk of disease is associated with BKV‐specific T‐cell dysfunction and that robust T‐cell responses play a major role in BKV infection control.^23^, ^24^ Broadening our understanding of T‐cell immunity against BKV is therefore of critical importance for improving outcomes in patients with BKV‐associated diseases. In this study, we therefore aimed to comprehensively profile T‐cell immunity against BKV in both healthy individuals and kidney transplant patients and have provided an in‐depth analysis of the antigenic immunodominance profile of BKV‐encoded antigens. We have also identified a large panel of novel antigenic determinants and demonstrated strong cross‐recognition of orthologous JCV peptides. Profiling of BKV‐specific T‐cell responses in renal transplant recipients revealed significant impairment of virus‐specific CD8^+^ and CD4^+^ T‐cell immunity in patients with high viraemia indicating a crucial role of these effector cells in controlling post‐transplant viral reactivation.

The overall T‐cell response to the BKV antigens showed an immunodominant response to the major capsid protein VP1 and both the T antigens (LTA and STA). A predominant CD4^+^ T‐cell response was directed towards VP1, LTA and STA, while a CD8^+^ T‐cell response was significantly higher for LTA and STA peptides with smaller magnitude responses to VP1. While the results in our study were somewhat discordant with previous studies that demonstrate immunodominant responses directed towards VP1,^25^, ^26^ these studies were limited in their analysis through their use of a restricted number of 9‐mer peptides.^11^, ^27^ The use of overlapping pools of 15‐mer peptides in the current study provides a more thorough and complete analysis, which leads to the identification of 20 CD8^+^ T‐cell epitopes and 10 CD4^+^ T‐cell epitopes covering a wide range of HLA types. Interestingly, a large proportion of these epitopes were cross‐reactive with JCV peptides, which highlight the potential use of these epitopes for future development of immunotherapeutic strategies which may be applicable for both BKV‐ and JCV‐associated complications.

Circulating BKV‐specific T cells in the peripheral blood are rare, most likely due to restriction of BKV infection to the kidney and genitourinary tissue and low and sporadic viral replication that requires few circulating memory T cells to control.^25^, ^28^ Despite their low frequency, BKV‐specific T cells seem to be very effective in controlling BKV infection particularly in asymptomatically infected healthy individuals.^9^ Our analysis of the functionality of circulating BKV‐specific T cells revealed a unique profile characterised by low expression of transcription factors T‐bet and Eomes and higher expression of CD27 and CD28. While this signature differs from that found in T cells specific for other persistent viruses, this finding is in line with previous observations by van Aalderen *et al*. showing a less differentiated central memory phenotype.^10^, ^11^, ^29^ While the history of primary infection with BKV is not well understood as it is acquired early in childhood and causes limited symptoms, reactivation of BKV in healthy individuals is likely less common than other latent viruses, such as CMV, suggesting a lower requirement for the maintenance of effector memory T cells in circulation. Consistent with this, it has been shown in various studies that antigen load and persistence influence the maintenance of the functional and phenotypic heterogeneity of T cells.^30^, ^31^, ^32^

Despite the presence of a less differentiated phenotype in BKV‐specific T cells, these antigen‐specific T cells retained the capacity to differentiate into effector cells following *in vitro* stimulation, characterised by enhanced T‐bet and granzyme B expression. However, this *in vitro* maturation was not associated with increased expression of Eomes or perforin, other key regulators of effector function. It remains to be determined what impact this lack of Eomes and perforin expression has upon immune control by BKV‐specific T cells, whether it is due to an intrinsic defect in these cells or whether it can be overcome by modifying the *in vitro* stimulation conditions. Despite this lower expression of perforin, BKV‐specific T cells efficiently killed antigen expression target cells, indicating they retain strong cytolytic potential. The *in vitro* maturation of BKV‐specific T cells was also associated with increased expression of cell migration markers such as CCR4, CD103, CD49d, CD11a, CD29 and CD18. Interestingly, previous observations have shown that circulating VP1‐specific T cells lack the expression of epithelial homing markers such as CCR4 and CCR9.^11^ Therefore, the promotion of an enhanced migratory ability following *in vitro* expansion could provide a strategy to improve T‐cell control in BKV‐associated diseases.

The data presented here strongly suggest that T cells directed against multiple BKV antigens may be essential in controlling BKV‐associated complications in transplant recipients. Furthermore, comprehensive mapping of CD4^+^ and CD8^+^ T‐cell epitopes will allow the development of better assays to monitor immunity to BKV, and the observed cross‐reactivity of T cells directed against BKV epitopes also extends the therapeutic potential of these T cells to other polyomaviruses such JCV. Identification of a broader range of T‐cell epitopes restricted through multiple HLA alleles from multiple BKV antigens significantly expands the potential target molecules for *in vitro* expansion of BKV‐reactive T cells for adoptive immunotherapy and the design of a prophylactic vaccine against BKV and JCV.

## Methods

### Study subjects

The study was performed according to the principles of the Declaration of Helsinki. It was approved by the QIMR Berghofer Medical Research Institute Human Research Ethics Committee and the Metro South Human Research Ethics Committee. All volunteers on the study provided written informed consent. Peripheral blood mononuclear cells (PBMC) were isolated from a cohort of 53 healthy individuals and 26 kidney transplant recipients (with or without the history of BKV reactivation) from the Princess Alexandra Hospital, Brisbane. The clinical characteristics of all the subjects included in the study are shown in Table 3. All subjects were HLA‐typed for this study.

**Table 3:** Clinical and demographical characteristics of the study subjects

Patient characteristics	KT recipients (*N* = 26)
Age (years), median (range)	53 (22–71)
Gender (male/female)	17 (M)/9 (F)
Time since transplant (months), mean (range)	10 (2–40)
Number of patient with BKV reactivation post‐transplant	14
Median BKV load (copies per mL serum), range	900 (16 −1.9E4) copies per mL
Pre‐transplant CMV status (% positive)	65%

### *In vitro* expansion of BKV‐specific T cells

Peripheral blood mononuclear cells from volunteers were washed and resuspended in RPMI‐1640 containing 10% foetal bovine serum (R10). The cells were then incubated with BKV overlapping peptide pools (OPPs) from the BKV‐encoded proteins LTA, STA, VP1, VP2 and VP3 at a concentration of 1 µg mL^−1^ and incubated at 37°C and 6.5% CO_2_ for an hour. The cells were then washed and cultured for 14 days in 24‐well plates incubated at 37°C and 6.5% CO_2_. These cultures were supplemented with R10 medium containing recombinant interleukin‐2 (IL‐2; Komtur Pharmaceuticals) at 20 IU mL^−1^ and TCGF from MLA144 cell line on day 2 and then supplemented with media containing TCGF and IL‐2 every 3 days thereafter. On day 14, T cells were counted using the trypan blue exclusion method and used for an IFN‐γ ICS assay, while the remaining cells were cryopreserved in liquid nitrogen. For gene expression and phenotypic analysis, T cells were stimulated with a pool of BKV‐specific defined peptide epitopes (Table 1) or a pool of CMV‐specific defined peptide epitopes described previously.^33^

### Intracellular cytokine analysis

Cultured T cells were incubated with antigens (including BKV‐OPPs, BKV peptide matrices, minimisation peptide and defined peptide epitopes) in R10 containing GolgiPlug (BD Pharmingen, San Diego, CA) alone for IFN‐γ analysis, and with GolgiStop (monensin; BD Pharmingen) and anti‐CD107a antibody for multiparametric analysis. After 4 h of incubation at 37°C and 6.5% CO_2_, the cells were washed with PBS containing 2% FBS (wash buffer) and the pellet was resuspended in wash buffer containing FITC‐conjugated anti‐CD4 and PerCP‐Cy5.5‐conjugated anti‐CD8 antibodies for IFN‐γ analysis, or with PerCP‐Cy5.5‐conjugated anti‐CD8 and PE‐Cy7‐conjugated anti‐CD4 for multiparametric analysis, and then incubated at 4°C for 30 min. Cells were then washed twice with PBS, fixed and permeabilised with Cytofix/Cytoperm solution (BD Pharmingen) for 20 min. Cells were then washed and incubated with PE‐anti‐IFN‐γ for IFN‐γ analysis or with PE‐conjugated anti‐IL‐2, Alexa Fluor 700‐conjugated anti‐IFN‐γ and APC‐conjugated anti‐TNF for multiparametric analysis (BD Pharmingen, San Diego) at 4°C for 30 min. Stained cells were washed twice with Perm/Wash buffer, resuspended in PBS containing 1% paraformaldehyde and acquired using a BD LSRFortessa. Post‐acquisition analysis was conducted using FlowJo software (TreeStar; FlowJo LLC, Ashland, OR).

### Gene expression analysis

BKV‐ or CMV‐specific T cells expanded from healthy individuals were labelled with MHC–peptide dextramers for 30 min at 4°C, before staining with anti‐CD8‐PerCP‐Cy5.5 (BD Biosciences), anti‐CD4‐FITC (BD Biosciences), anti‐CD3‐PE and LIVE/DEAD Fixable Aqua Dead Cell Stain (Thermo Fisher) for 20 min at room temperature. Cells were washed in PBS and resuspended in PBS at approximately 1 × 10^7^ cells mL^−1^. Using a FACSAria III cell sorter (BD Biosciences, San Diego), approximately 10 000 BKV‐specific or CMV‐specific T cells were sorted into PBS containing 2% FCS and then lysed using cell lysis buffer (QIAGEN, Melbourne, Australia). Gene expression analysis of the cell lysate was conducted on the NanoString nCounter gene expression platform (NanoString Technologies, Bio‐Strategy, Brisbane, Australia). A custom code set consisting of a 136‐gene panel related to T‐cell biology, immune regulation and immune cellular markers was used. For each sample, 5 μL of lysate was mixed with a 3′ biotinylated capture probe and a 5′ reporter probe tagged with a fluorescent barcode from the custom gene expression code set. Probes and target transcripts were hybridised at 65°C for 12–16 h as per the manufacturer's recommendations. Hybridised samples were run on the NanoString nCounter preparation station using the high‐sensitivity protocol, in which excess capture and reporter probes were removed and transcript‐specific ternary complexes were immobilised on a streptavidin‐coated cartridge. The samples were scanned at maximum scan resolution on the nCounter Digital Analyzer (NanoString Technologies, Bio‐Strategy, USA). Gene expression data for each individual sample were normalised by housekeeping normalisation. The log_10_ count of each gene on the platform was normalised by subtracting of the arithmetic mean of the log_10_ counts of the housekeeping genes. Differentially expressed genes between CMV and BKV were identified using *t*‐test with multiple testing correction (corrected *P*‐value < 0.05). Pathway enrichment and gene ontology analysis was performed using the InnateDB^34^ platform, which identifies the top KEGG, Reactome and GO Molecular Function and Biological Process terms enriched among the input set of genes.

### T‐cell immunophenotyping

To label cell surface antigens, BKV‐ or CMV‐specific T cells were washed once in wash buffer, incubated with fluorochrome‐conjugated antibodies for 15–20 min at 4°C, washed twice in wash buffer and then fixed in 1% paraformaldehyde (Sigma‐Aldrich, Castle Hill, NSW). The antibodies used included CD3, CD4, CD8, CD27, CD57, CD95, CD45RA and CD28 conjugated to fluorescein isothiocyanate (FITC), phycoerythrin (PE), peridinin–chlorophyll (PerCP), peridinin–chlorophyll with cyanine 5.5 (PerCP‐Cy5.5), allophycocyanin (APC) or Alexa Fluor 700. Antibodies were purchased from BD Pharmingen (North Ryde, NSW) or eBioscience (San Diego, CA). For co‐staining with MHC–peptide multimers, cells were first incubated with multimer for 20 min at 4°C, and then, surface antibodies were added and incubated for a further 15–20 min at 4°C. The cells were washed twice with PBS–2% FBS and fixed in 1% paraformaldehyde. Cells were acquired on BD LSRFortessa using FACSDiva software (Becton Dickinson, San Diego, CA). Results were analysed using FlowJo software (TreeStar, Ashland, OR). To define phenotypically distinct T‐cell populations, Boolean analysis was performed using FlowJo software.

### Bead‐based enrichment of virus‐specific T cells

To enrich BKV‐specific T cells from the PBMC, about 10 million cells were washed and resuspended in 100 μL of wash buffer (PBS with 0.5% BSA and 2 mm EDTA) and incubated with 10 μL of APC‐MHC dextramers. The cell suspension was then washed with wash buffer and incubated with 20 μL of anti‐APC microbeads (Miltenyi Biotech). The cell suspension was then washed and passed through LS MACS column. The APC‐conjugated cells bind to the column, and the flow‐through was discarded. The APC‐positive cells were then collected in a fresh tube by syringe‐flushing the column. The collected cells were used for further staining to be characterised in flow cytometer.

### BKV cell line development

BKV‐ST antigen and BKV‐VP1 antigen constructs in pUC vector were synthesised at GenScript Incorporation (USA). BKV antigens were then cloned into pEGFP‐N1 vector, and the resulting plasmids were named pGFP‐ST and pGFP‐VP1. The constructs were then transfected into HEK293 cells using Lipofectamine 3000 (Life Technologies). The constructs were confirmed for the expression of BKV antigens by Western blot. The resulted HEK293 cells expressing BKV antigens were named VP1‐HEK293 cells and ST‐HEK293 cells. These cells were used as target cells in the T‐cell killing assay.

### Real‐time T‐cell killing assay

The xCELLigence RTCA MP Instrument (ACEA Biosciences) was used for measuring the T‐cell killing of the BKV antigen‐expressed cells. ST‐HEK293 cells were first seeded at a density of 60 000 cells per well of the E‐plate to a total volume of 150 μL with DMEM–10% FCS media. The E‐plates were kept in the incubator for 30 min and then transferred to the RTCA instrument inside the tissue culture incubator. The data were continually measured at 30‐min intervals for the entire experiment. At the time of addition of T cells, which is approximately 24 h after seeding, the data acquisition was paused and the E‐plate was transferred to the laminar hood. A total of 100 μL of media was removed from each of the wells and was added with T cells at an effector‐to‐target ratio of 10:1 in a volume of 100 μL. The E‐plates were kept in the laminar hood for 30 min before being transferred to the RTCA instrument. Data acquisition was resumed, and the cell index value was measured for 72 h.

### Statistical analysis

Statistical analysis was performed using Prism 9 software (GraphPad Software). Statistical significance was determined using the nonparametric Mann–Whitney *U*‐test for single comparisons. Comparisons were considered statistically significant at *P*‐value < 0.05.

## Conflict of interest

GA, BTA and RK are listed as inventors on international patent application that includes multiple BKV epitopes. RK and CS are consultants for Atara Biotherapeutics and received research support from Atara Biotherapeutics.

## Author contributions

RK and CS designed the study. GA conducted most of the experimental studies. DC, SS and BTA contributed to data analysis. RSF contributed towards the recruitment of renal transplant patients and clinical monitoring. All authors contributed to the drafting of the manuscript and approved the final version of the manuscript.

## Data Availability

All requests to access the data included in this manuscript can be made in writing to Professor Rajiv Khanna (rajiv.khanna@qimr.edu.au).
