# Supplementary figures and images for: Proteome‐wide analysis of T‐cell response to BK polyomavirus in healthy virus carriers and kidney transplant recipients reveals a unique transcriptional and functional profile
Source: Clin Transl Immunology. 2020 Jan 14;9(1):e01102. doi: 10.1002/cti2.1102 (PMC6960379; doi:10.1002/cti2.1102)

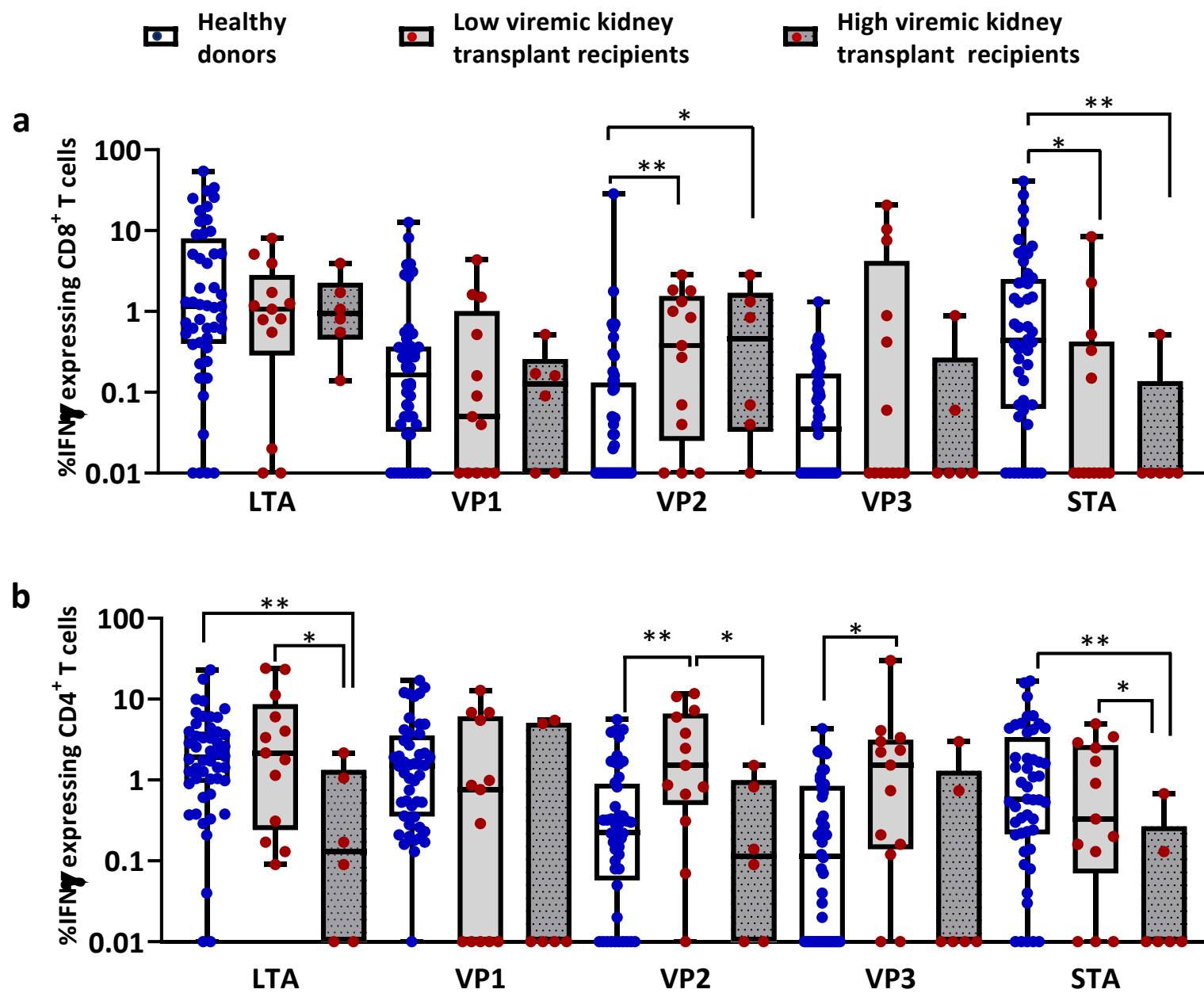

Fig 1

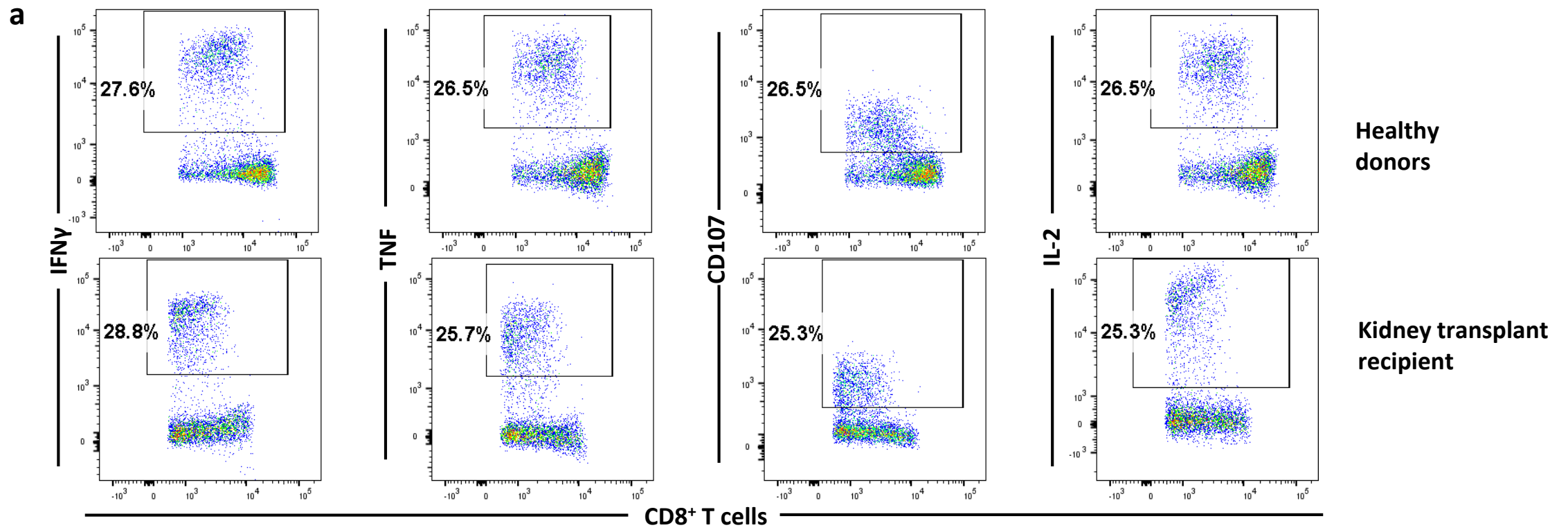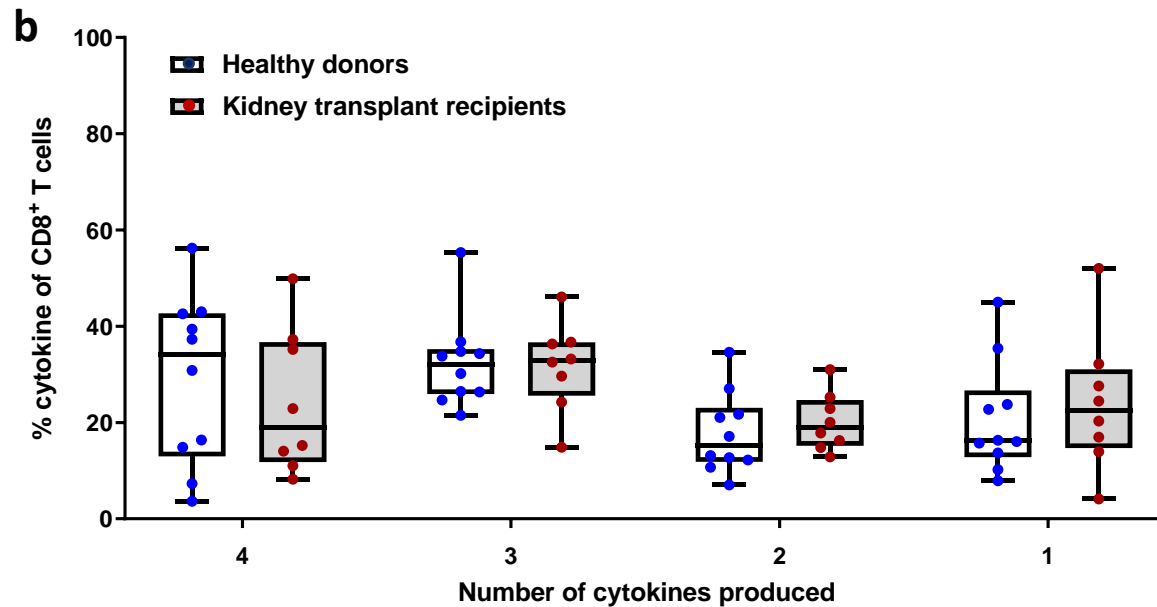

**Fig 2**

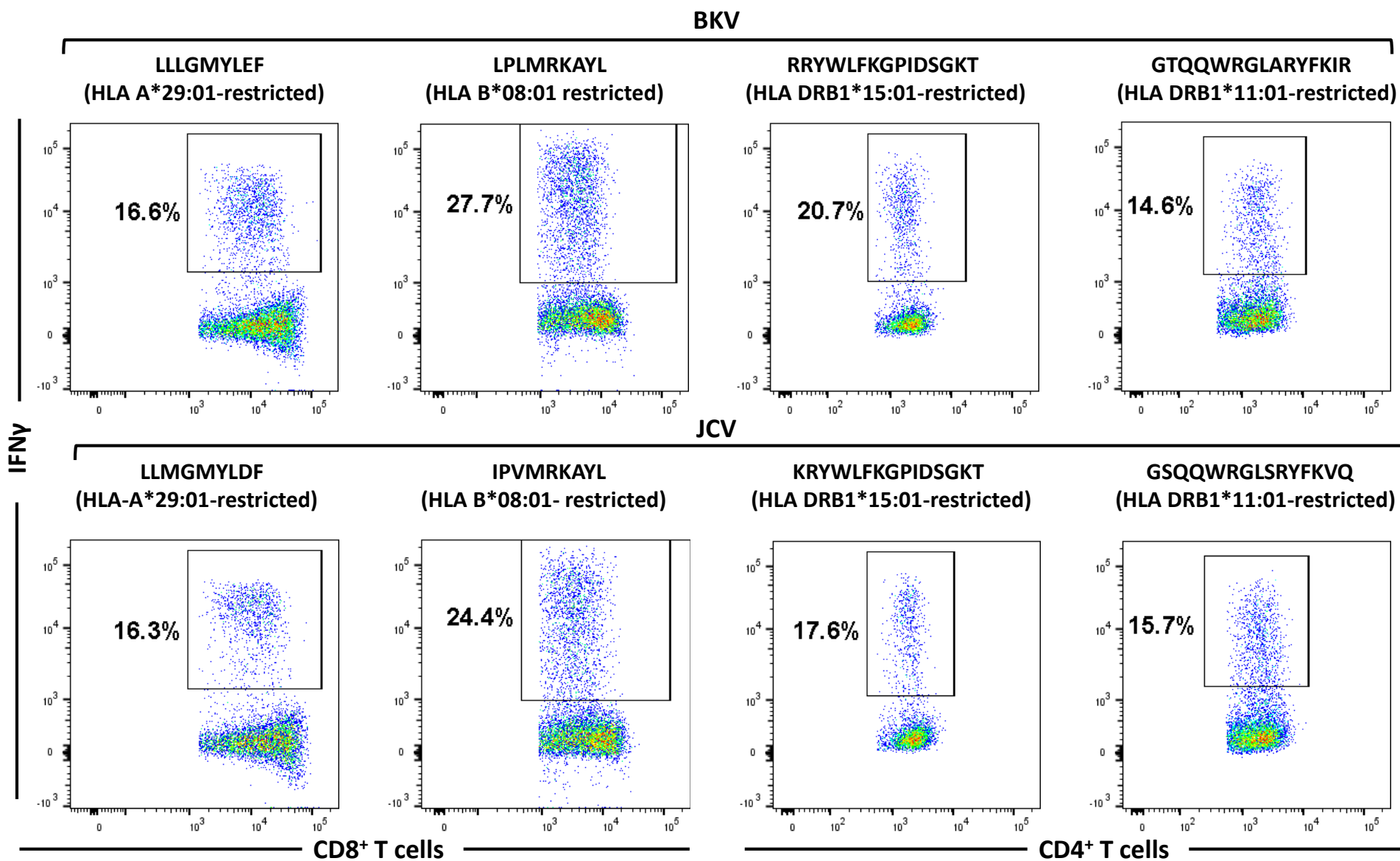

**Fig 3**

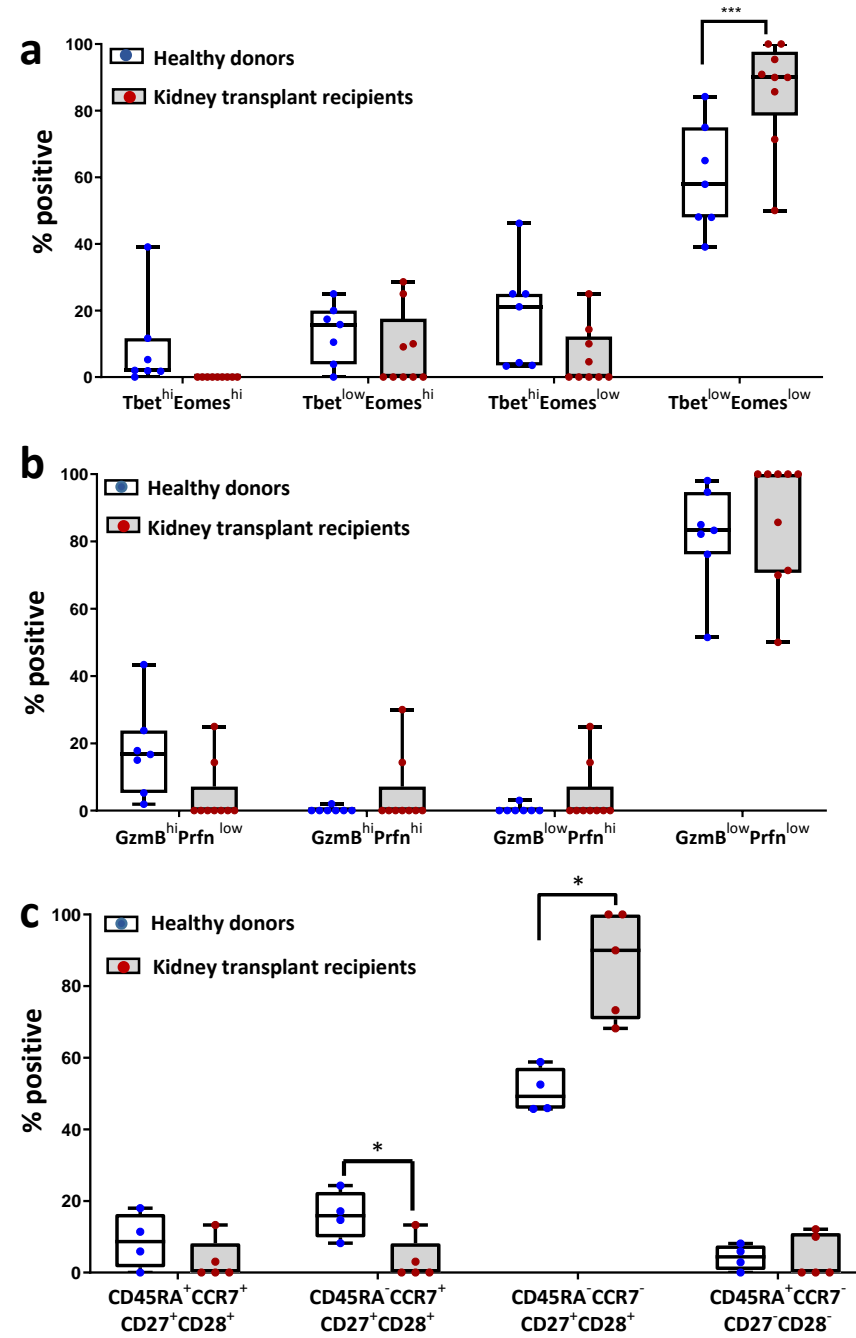

**Fig 4**

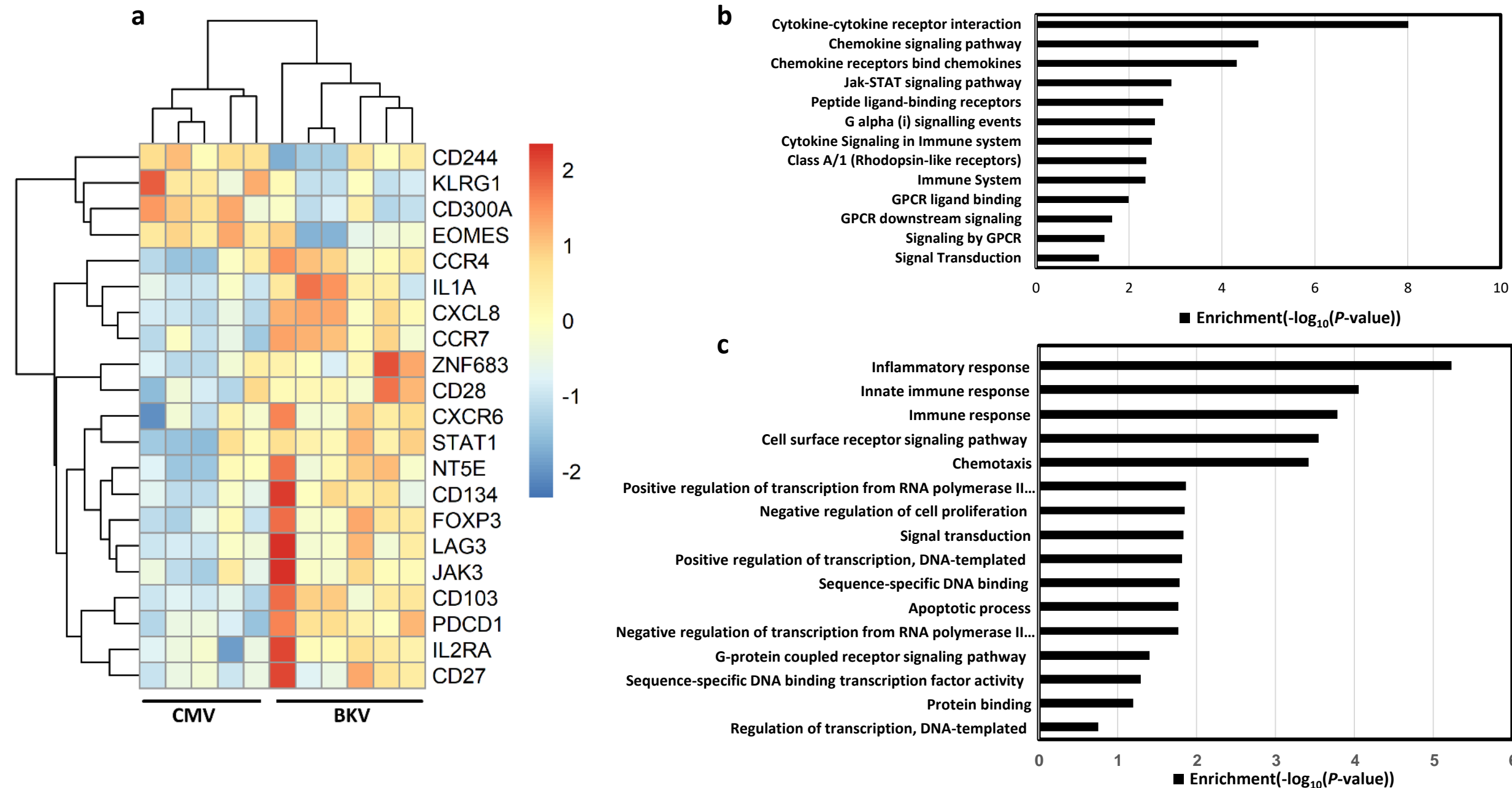

**Fig 5**

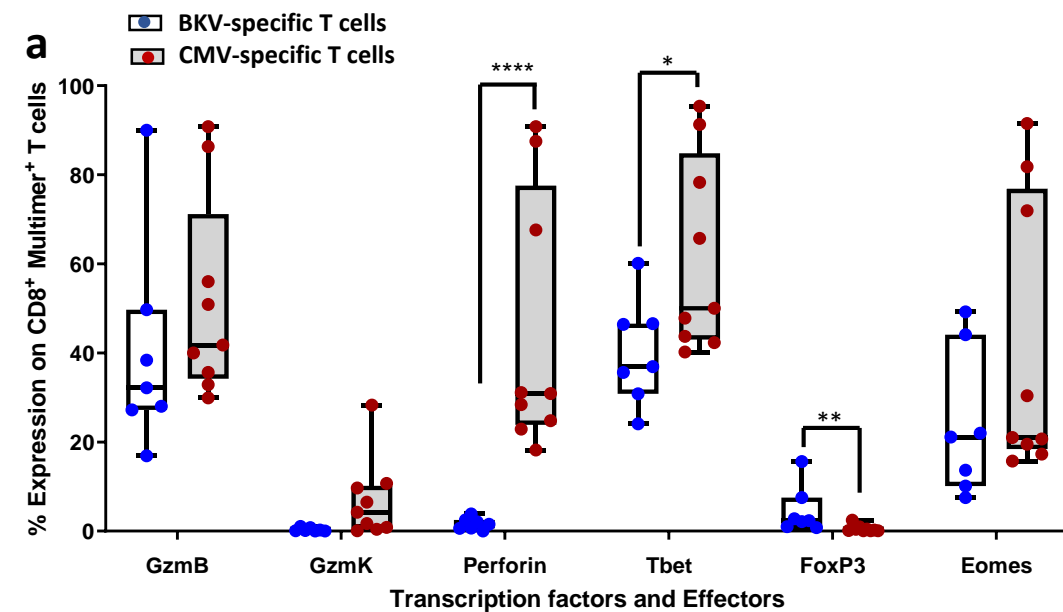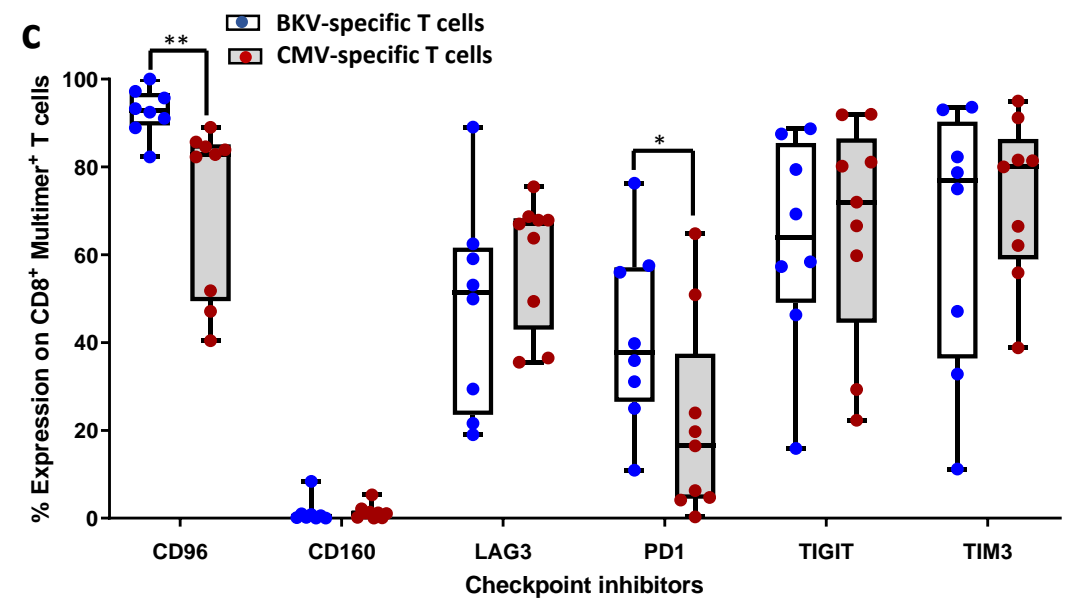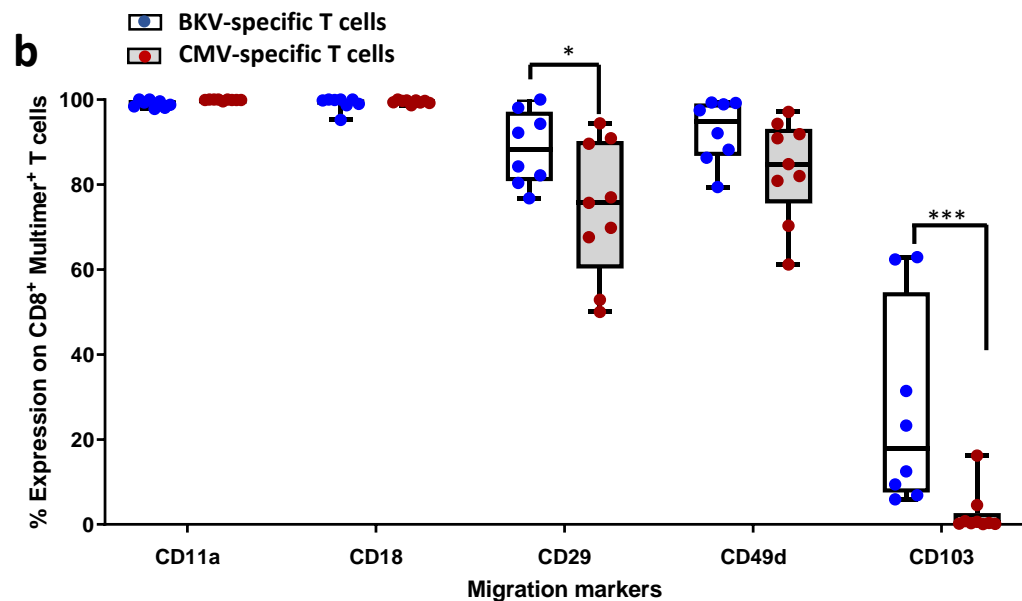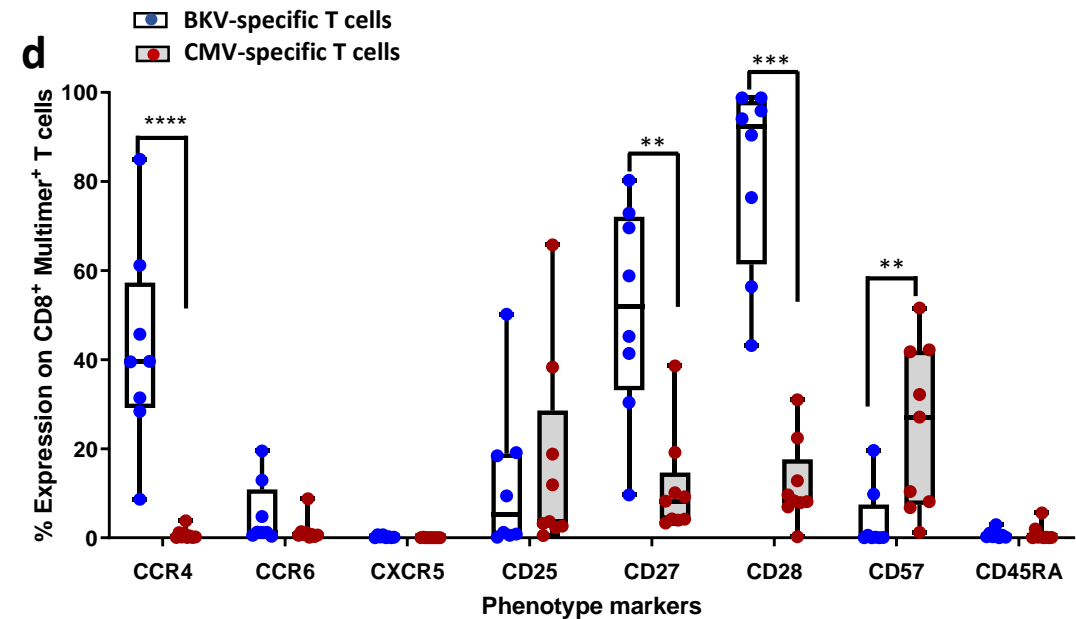

**Fig 6**

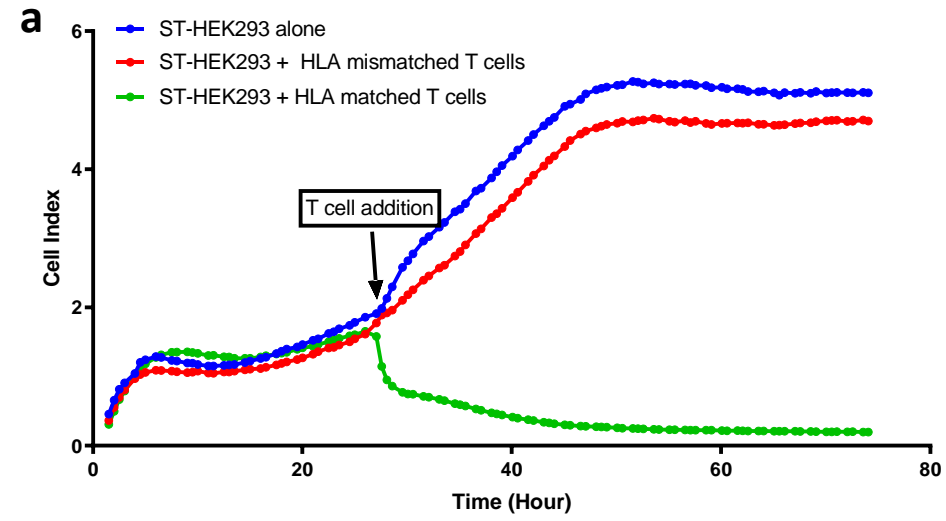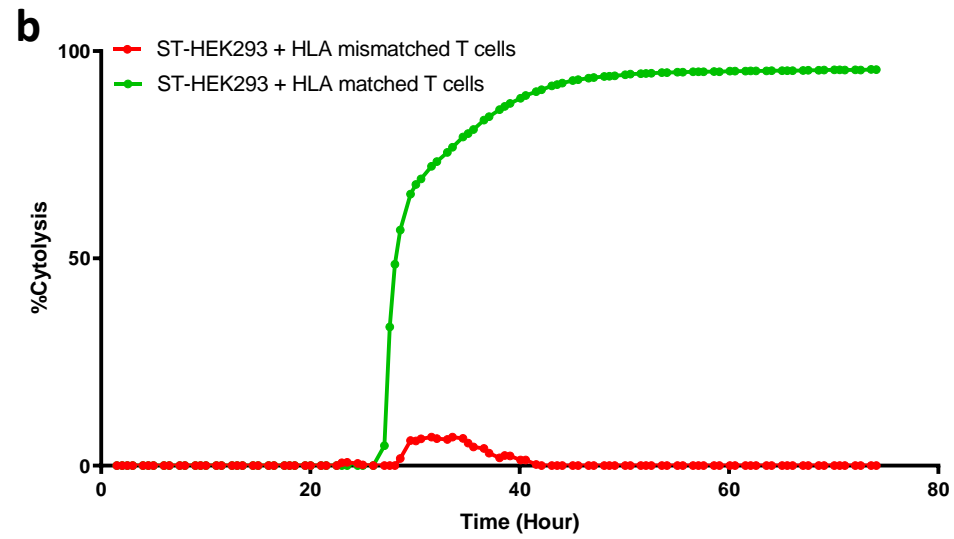

**Fig 7**

# Supplemental Figures

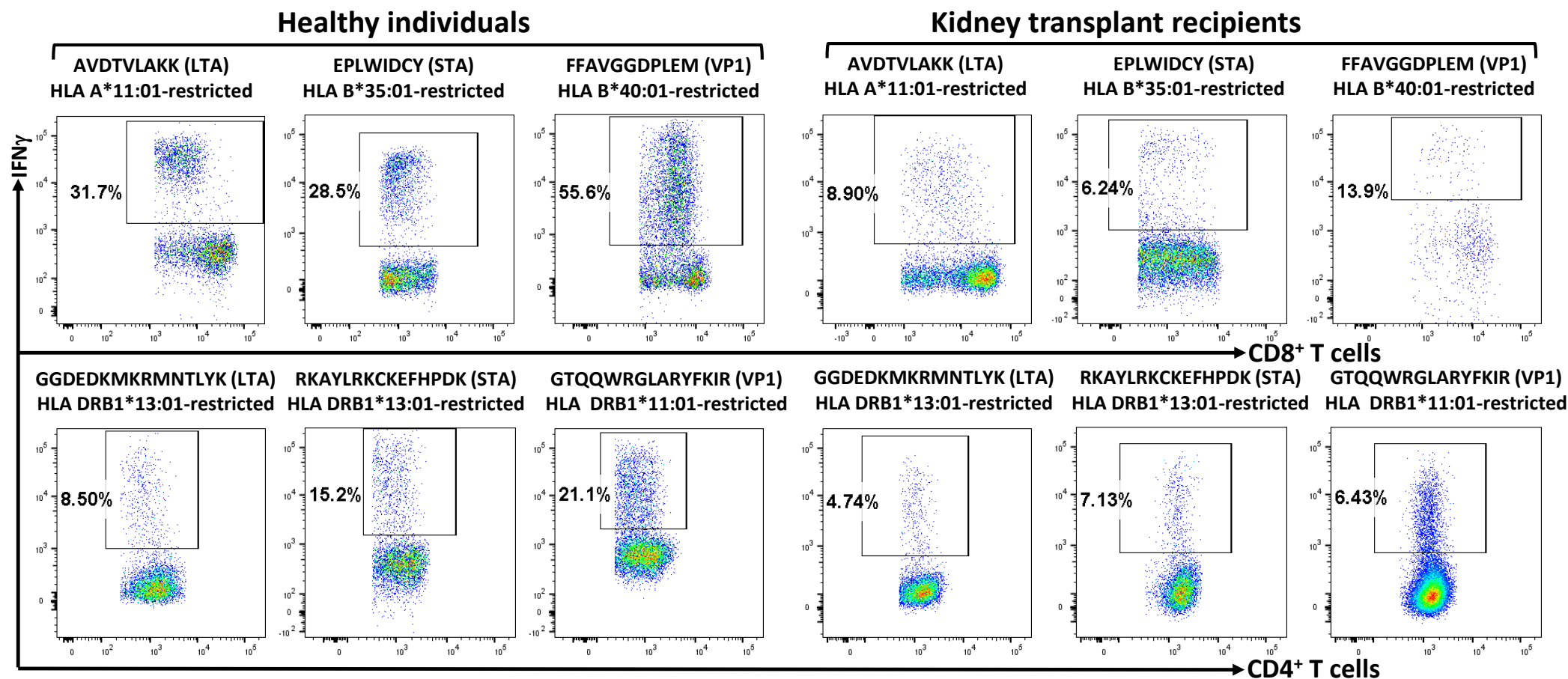

**Supplemental Fig 1**

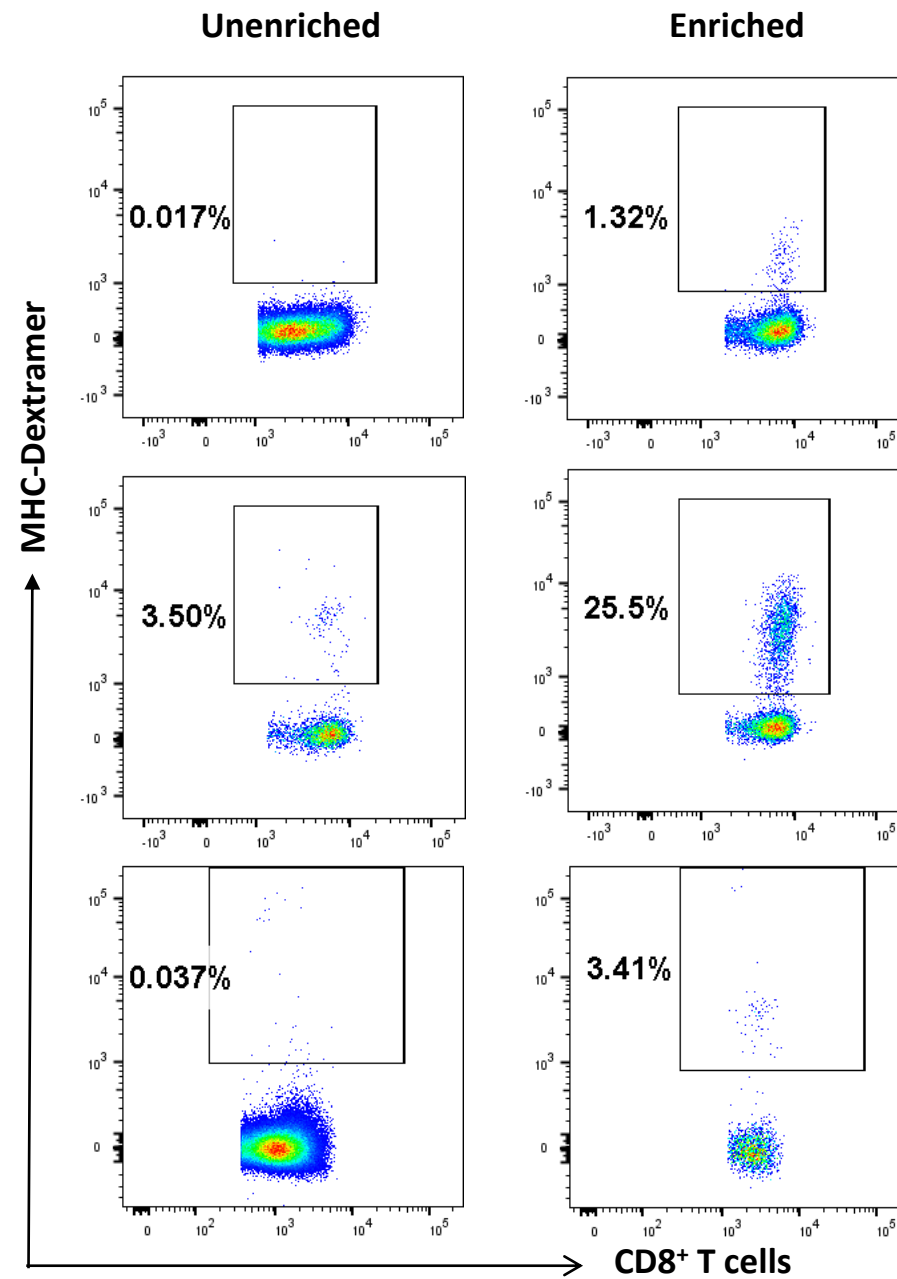

Supplemental Fig 2

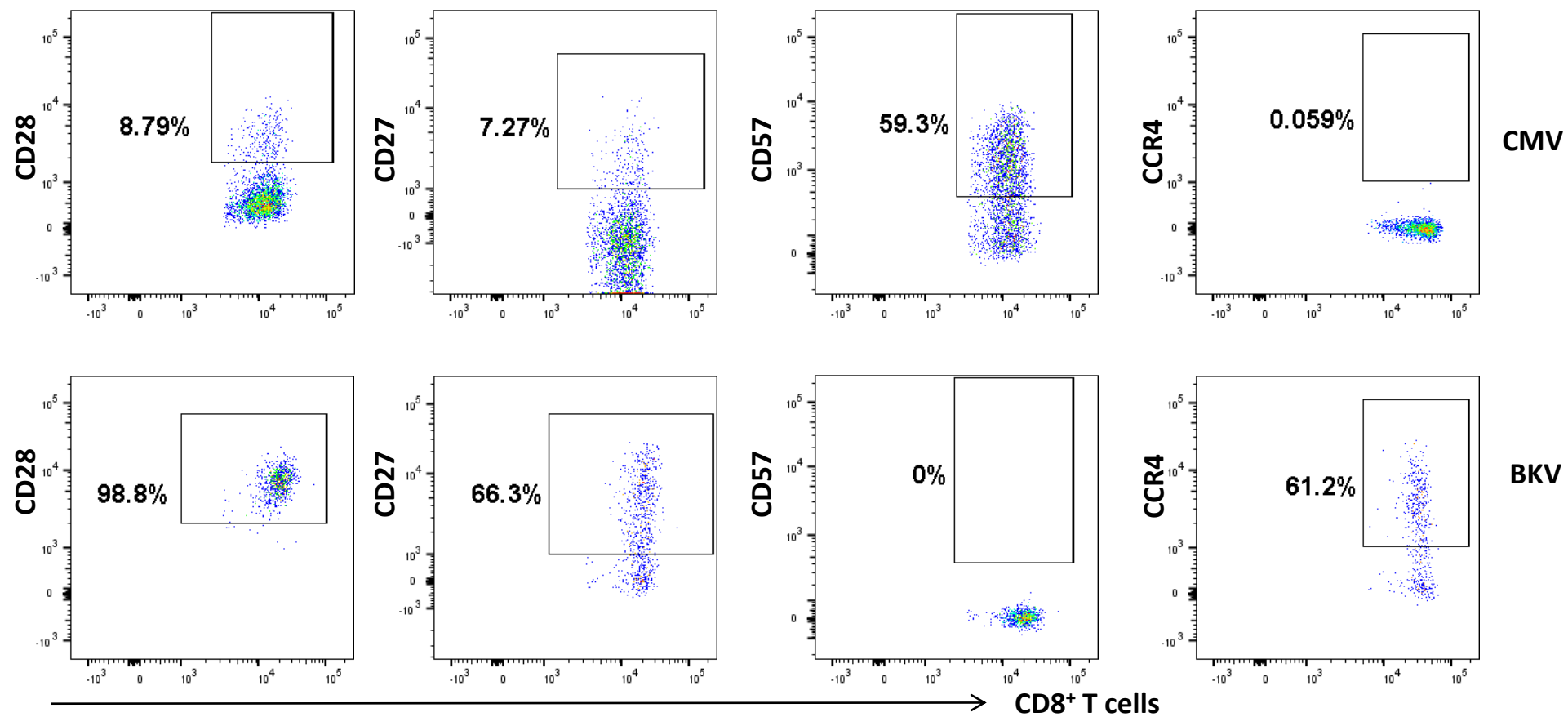

Supplement: Supplementary file 1 [file CTI2-9-e01102-s001.pdf]
